# A Review and Criticism on the Transactions of the Dental Association of Maryland and District of Columbia for the Year 1878

**Published:** 1879-08

**Authors:** J. J. Caldwell, Martin P. Scott

**Affiliations:** Baltimore; 68 W. Fayette Street, Baltimore, Md.


### A REVIEW AND CRITICISM ON THE TRANSACTIONS OF THE DENTAL ASSOCIATION OF MARYLAND AND DISTRICT OF COLUMBIA, FOR THE YEAR 1878.

### ANÆSTHESIA AND ANÆSTHETICS.

*Address by J. J. Caldwell, M. D., Baltimore.*

I have read with much pleasure Dr. Caldwell's address, and received much instruction. It is a well considered and thoughtful resume of the subject of Anæsthetics to the present time. Not only has he presented the views of the ablest writers on the subject, but has added his own comments on their physiological action, which, if my esteemed brother will permit me to say, are worthy to rank with the best. He justly gives the first rank to chloroform in all operations, except those in dental surgery, which require the sitting posture. Chloroform is better than ether or any of its mixtures. Chloroform is king. In the chain of criticism we avail ourselves of the privilege and speak *ex cathedra.*

The mode of administration is dwelt upon at sufficient length, where he says the patient must be recumbent, the stomach empty and sufficient amount of atmospheric air admitted along with the agent. We are sorry he did not condemn in stronger language the pernicious and dangerous practice of overpowering the patient at once with a large quantity of chloroform; better garotte to insensibility and proceed with the operation.

The precautions against swallowing the tongue (a kind of feli disc) are made s ıfficiently prominent, as well as the physiological condition upon which the accident supervenes. It is regretted that he did not analyze with sufficient exhaustiveness (excuse the word) the causes of death, as by coma, asphyxia and the like, as in that event he would have traced, with but few exceptions, death to failure to admit atmospheric air in sufficient quantities.

I have administered chloroform many hundred times, have witnessed its administration in the hospitals of Paris, in the field and in hospitals during the late civil war, and have yet to see the least unpleasantness follow where this precaution has been used.

Two points should be borne in mind, the carbonized condition of the blood, and the specific action of the agent upon the brain and other nerve centers. People vary in susceptibility to its influence. The negro succumbs more readily than the white race, a phy.iological fact to which I would invite the attention of those who insists upon negro equality.

We take this occasion to say that the views of Dr. Chisholm in reference to the necessity of bringing the patient fully under the influence of chloroform before commencing an operation, to render the reflex motor system insensible as the sentient nervous centers, have our hearty concurrence. Shock is thus avoided, which we know is so often fatal.

Chloroform has not been considered by the Doctor or by any of the authors cited as a therapeutic agent in the treatment of disease. A substance which acts so powerfully upon every part of the nervous system, centrically and excentric, must be a most important remedial agent in a large number of diseases, for there is scarcely one in which the nervous system is not more or less concerned. As early as 1856 I used it in treatment of colic with magical effect. During the late war colic was very common among our soldiers returned from northern prisons. I used it in a number of severe cases and always with most satisfactory results. It does more than relieve, temporarily, pain, for in many cases the pain does not recur after the effects of the chloroform have apparently subsided. In passage of calculus from kidney it is emphatically *the remedy*, at least in my hands, I have had but little trouble with this affection. I administer by inhalation. I have never used it but once in cold stage of intermittent. The patient had been shaking for two hours in a way I never saw any one else shake; black under the eyes; an Arkansas shake. I thought she would die. I gave chloroform (inhalation) the paroxysms ceased instantly, it did not return. A slight fever followed. I used it on the same person a second time under the same circumstances. On this occasion the shivering and shaking returned. I repeated chloroform, arrest, recurrence. At the end of about twenty minutes, being under it influence from time to time, the cold stage was arrested. I would suggest the use of chloroform in intermittent and malarial fevers in their outset as a curative agent. Would that some substitute might be discovered for quinine, to deprive those harpies of Philadelphia of their blood money, wrung from the sick poor of our country through the instrumentality of an iniquitous tariff.

Chloroform is worthy of a trial in that scourge if the tropics and our southern country yellow fever. I suggest it in these cases upon the idea of the same theory of such diseases, the most rational yet put forth of their pathology.

I trust, therefore, that our friend and brother at some future time will discuss the use of chloroform as applied to treatment of diseases. Internally I would suggest it as a remedy for all parisites. It will kill the bott, so fatal to the horse. We must be permitted to thank the Doctor for that part of his address in which he considers the methods to resuscitate from the effects of chloroform. He has given prominence to the use of Nitrite of amyl., (see Dabney). Interesting experiments are cited on dogs, showing its powerful effects, due, as he believes, to its action on the heart, conclusions which are strengthened by two cases of resuscitation, reported by Dr. R. W. Nelson, of Virginia. It is worthy of remark that of the four dogs experimented upon, the only one not revived by the Nitrite of amyl. was the one to whom chloroform had been given *unmixed with atmospheric air.*

Artificial respiration is also recommended. Dr. Cutter, of Boston, has furnished what has been so much needed, a simple mechanical contrivance to pump air or oxygen into the lungs and pump it out. "To pump air in and out of the body without any pressure on the chest—to breathe life into persons."

But I would ask especial attention to that part of Dr. C.'s essay which treats of the application of Faradaiac electricity. Some of the experiments cited upon the pneumograstic nerve, after its division, in which electricity supplied the place of nerve force in digestion, are most interesting. It has long been known that electricity is a powerful nervous stimulant, but here we have the fact arrived at of identity of nerve power aud electric force. The process of digestion being carried on by substituting a battery for the nerve centers of the pneumogastic nerves.

Such being the case there can not rest a doubt that in the battery we have the most powerful and efficient means of restoring persons to life when every other known agent would fail. As merely an aid, a stimulant, its powerful effects are recognized, but if indeed the subtle agent we call electricity be the same as nerve force we shall have attained a point in physiology which may enable us to unravel the problem of life. Setting aside all that our confrere has said in regard to chloroform, its physiological action, its mode of administration, the means of restoration from its pernicious effects, we regard his suggestion of electricity as the most potent remedy, and opening a new field for its application as a therapeutic agent in the treatment of disease. We trust that Dr. Caldwell will again give us the benefit of his experience and reflection upon Faradaiac electricity.

In conclusion we would recommend all who desire, in brief to review the subject of anæsthetics, to consult Dr. Caldwell's admirablc resume of the subject, as set forth in the transactions of the Dental Association of the State of Maryland and District of Columbia for the year 1878.

I can not close my remarks without a word to the dental profession. I am happy to congratulate them upon the great advance and upward progress of the profession of dentistry. Like surgery it long remained a purely mechanical art. Time was when surgery was a part of the barber's calling. Time was when medicine was so low that the priest was inhibited from its practice, although in England Macauley tells us they (the priests) sometimes curried the coach horses and were prohibited from marrying the servant girls. The progress of intellectual development has taken all these high callings from their menial condition. The exercises the noblest profession of the world; he is the minister and interpreter of God's Holy Word. Next to his is the mission of those who minister to the sick in body. Dentistry must now take its place as a branch of surgery. One of their schools has set us a noble example, by which great reforms may beeffected in medical education, so much needed in these days of medical multiplication. That proficiency, not length of time of study, should be the test demanded for graduation, and that the teachers should never be permitted to examine candidates or grant diplomas.

MARTIN P. SCOTT, M. D.,
68 W. Fayette Street, Baltimore, Md.

